# Upper critical magnetic field in NbRe and NbReN micrometric strips

**DOI:** 10.3762/bjnano.14.5

**Published:** 2023-01-05

**Authors:** Zahra Makhdoumi Kakhaki, Antonio Leo, Federico Chianese, Loredana Parlato, Giovanni Piero Pepe, Angela Nigro, Carla Cirillo, Carmine Attanasio

**Affiliations:** 1 Dipartimento di Fisica “E. R. Caianiello”, Università degli Studi di Salerno, I-84084 Fisciano (Sa), Italy; 2 CNR-SPIN, c/o Università degli Studi di Salerno, I-84084 Fisciano (Sa), Italy; 3 Dipartimento di Fisica “E. Pancini”, Università degli Studi di Napoli Federico II, I-80125 Napoli, Italy

**Keywords:** non-centrosymmetric superconductors, Pauli and orbital contribution, upper critical fields, Werthamer–Helfand–Hohenberg theory

## Abstract

Non-centrosymmetric superconductors have recently received significant interest due to their intriguing physical properties such as multigap and nodal superconductivity, helical vortex states, as well as non-trivial topological effects. Moreover, large values of the upper critical magnetic field have been reported in these materials. Here, we focus on the study of the temperature dependence of the perpendicular magnetic field of NbRe and NbReN films patterned in micrometric strips. The experimental data are studied within the Werthamer–Helfand–Hohenberg theory, which considers both orbital and Zeeman pair breaking. The analysis of the results shows different behavior for the two materials with a Pauli contribution relevant only in the case of NbReN.

## Introduction

Superconducting films of NbRe and NbReN have recently received great attention in the field of low-temperature electronics as suitable candidates for the realization of fast superconducting nanowire single-photon detectors (SNSPDs) [1–4]. Apart from the reduced values of the superconducting gap and short quasi-particle relaxation times [5], the property that makes these materials appropriate to be used as SNSPDs is the high value of the electrical resistivity [6]. This feature is related to the polycrystalline or amorphous nature of these materials when deposited in a thin-film form [4,7–8]. In addition to the applicative interest, the study of these materials is relevant from a fundamental point of view. Nb*_x_*Re_1−_*_x_* (NbRe) for 0.13 ≤ *x* ≤ 0.38 crystallizes in the non-centrosymmetric Ti_5_Re_24_-type structure with the space group 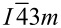 (No. 217) [9–12]. This non-centrosymmetric nature of the material leads to intriguing and unconventional physical properties such as the time-reversal symmetry breaking observed with muon-spin rotation and relaxation studies [13] and large values of the upper critical magnetic fields [11,14], which are above the Pauli paramagnetic limit [15–16]. In the case of thin films, the structure of NbRe is polycrystalline with grains of small dimensions, typically of the order of 2–3 nm [7–8,17]. NbReN films also present a polycrystalline nature with a moderate texture. In this case, it was possible to interpret the results of the structural characterization only by assuming for NbReN the same unit cell as that of NbRe [4]. However, detailed knowledge of its structural and microscopic properties is still lacking. Finally, while the morphological properties are similar to those of NbRe films [18], the values of the electrical resistivity stand slightly higher with respect to NbRe films [4,7,18].

The value of the upper critical magnetic field is a fundamental quantity that gives a measure of the robustness of the superconductivity in a specific material. For a standard BCS *s*-wave type-II superconductor, Werthamer, Helfand, and Hohenberg (WHH) have calculated the temperature dependence of the critical field [19]. In this model, two mechanisms are responsible for the breaking of the superconductivity and both contribute to the behavior of *H*_c2_(*T*), namely the orbital and the paramagnetic effect. While the former is due to the Lorentz force acting on electrons of the Cooper pairs with opposite momentum, the latter is related to splitting of the spin-singlet pairs because of the Zeeman effect. Accordingly, paramagnetic pair-breaking effects are absent in spin-triplet superconductors. The Pauli limiting field is given by 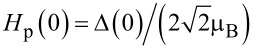, where Δ(0) is the superconducting energy gap at zero temperature and μ_B_ is the Bohr magneton [15]. For weakly coupled BCS superconductors it is *H*_p_(0) [T] ≈ 1.84*T*_c_ [K]. In the dirty limit, the orbital limit at zero temperature is given by 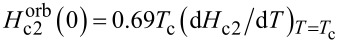 [19]. Close to the critical temperature, *T*_c_, the contribution of the orbital term dominates in determining the value of *H*_c2_, at low temperatures and high fields, the Pauli term is predominant [20–21]. In general, the relative weight of the two effects is given in the theory by the Maki parameter 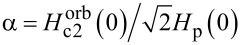 [19,22]. Alternatively, it could be convenient to express α in terms of normal-state properties [19,22–23]. In this case, α = 3/(2*k*_F_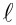) [24] where *k*_F_ is the Fermi wave vector and 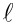 is the mean free path. If spin–orbit scattering is also taken into account, another quantity, λ_so_ (the spin–orbit parameter), is introduced in the theory [19]. The effect of λ_so_ is to soften the role of α in determining the values of *H*_c2_(*T*) [19]. When λ_so_ = 0, it is 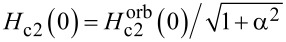 [23]. The WHH theory has been used to interpret the temperature dependence of the critical fields of several superconductors [20,24–36], including non-centrosymmetric materials for which experiments give contradictory results [14,37–43]. In particular, values of the critical field larger than *H*_p_(0) have been experimentally observed. These results, which cannot be described by the WHH model, are interpreted as an indication of the presence of an unconventional superconducting pairing in the material.

In this paper, we measure the temperature dependence of the perperpendicular, *H*_c2⟂_(*T*), and parallel, *H*_c2∥_(*T*), critical field of NbRe and NbReN microstrips. The behavior of *H*_c2⟂_(*T*) is analyzed in the framework of the WHH theory. We find that while for NbRe the value of the critical field is purely orbital-limited, in the case of NbReN, the effect of the Pauli contribution plays a relevant role in the temperature dependence of *H*_c2⟂_. We have not observed a positive curvature of *H*_c2⟂_(*T*) near *T*_c_ as it was in the case of two-band superconductivity [44] or proximity-coupled superconducting systems [45–46].

## Experimental

NbRe and NbReN films were sputtered on oxidized Si substrates in a UHV dc magnetron system with base pressure of 1 × 10^−8^ mbar. The films were deposited at room temperature from a stoichiometric NbRe (Nb_0.18_Re_0.82_) 99.95% pure target of 5 cm diameter at a power of 350 W. NbRe films, 8 nm thick, were grown at a Ar pressure of 4 μbar, which resulted in a deposition rate of 0.3 nm/s. NbReN films, 10 nm thick, were reactively sputtered in a mixture of inert Ar (85%) and reactive N_2_ (15%) gas at a total pressure of 3.5 μbar at a rate of 0.36 nm/s. NbRe films were patterned by standard optical lithography and lift-off procedures to realize a Hall bar geometry of width *w* = 10 μm and a distance between the voltage contacts of *L* = 90 μm. The NbReN films were structured by using direct laser writer exposure followed by argon ion etching into constriction-type bridges with *w* = 2 μm and *L* = 700 μm. Further details on the fabrication procedure of the films are reported elsewhere [4,8].

The superconducting properties have been analyzed by electrical resistance measurements using a standard four-probe technique in a Cryogenic Ltd. CFM9T cryogen-free system. The microstrips were biased with a current of *I*_b_ = 10 μA. During the measurements, the error on the temperature value was less than 10 mK. The superconducting *H*–*T* phase diagrams were obtained by measuring the resistive transitions in the presence of the magnetic field applied perpendicularly or parallely to the surface of the samples. For each field, the value of *T*_c_ was determined using the 50% *R*_N_ criterion, where *R*_N_ is the value of the normal-state resistance at 10 K.

## Results and Discussion

**Figure 1:**
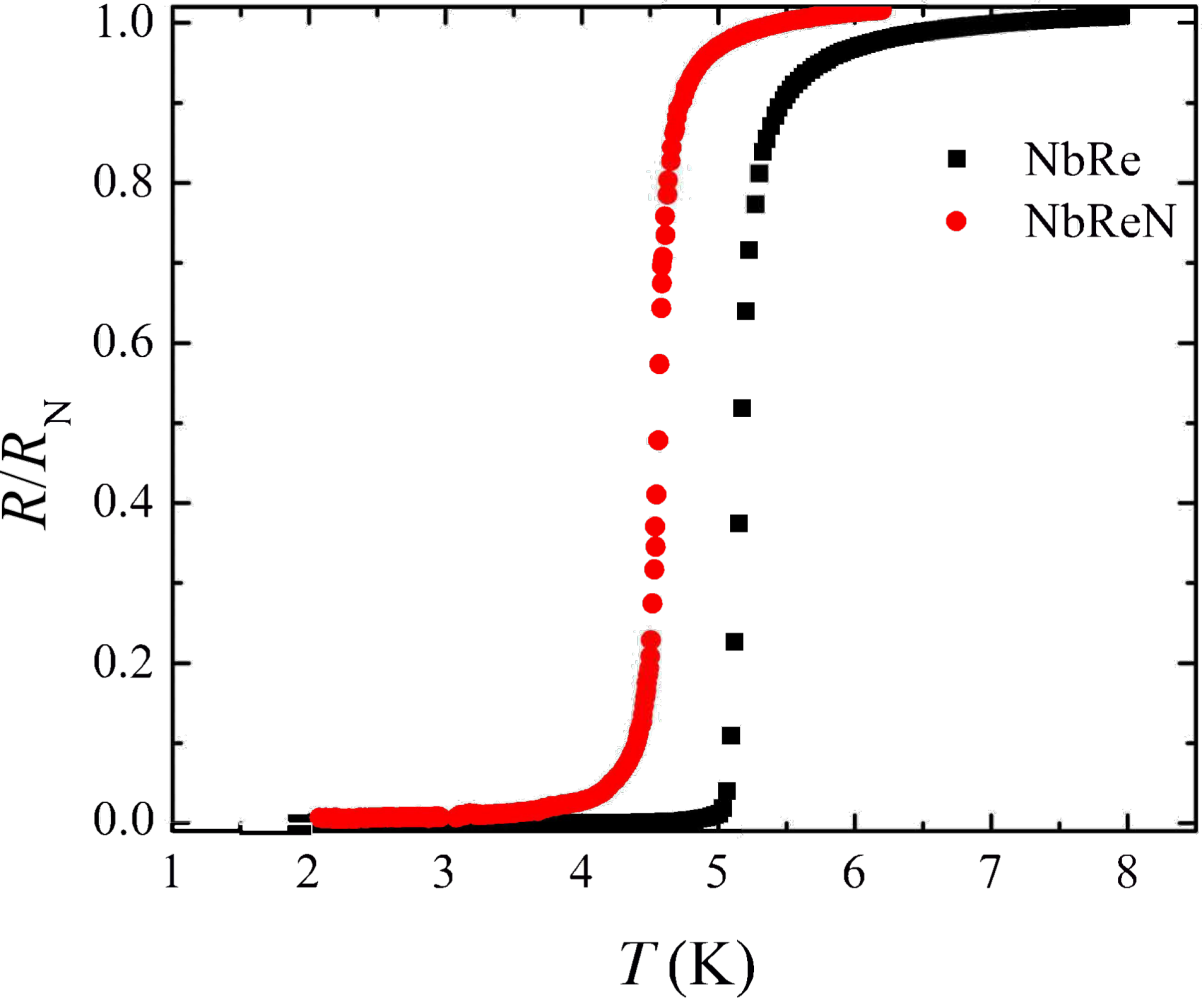
Resistive transition in zero magnetic field of the NbRe (black squares) and NbReN (red circles) microstrips.

Figure 1 displays the normalized resistive transitions in zero magnetic field of the NbRe and NbReN microstrips. The critical temperature, the low-temperature resistivity, and the residual resistivity ratio (RRR) are reported for both microstrips in Table 1. The RRR is defined as the ratio of the resistivity at room temperature and at 10 K, that is, RRR = ρ_300K_/ρ_10K_ = *R*_300K_/*R*_10K_. The values of *T*_c_ are not significantly smaller than the values measured on unpatterned films of the same thickness [4,8]. The rounding present at the onset of both the curves is due to the paraconductivity phenomenon, whose nature has been analyzed in detail in the case of unstructured NbReN films of different thickness [4].

**Table 1:** Parameter values of NbRe and NbReN microstrips.

Parameters	NbRe	NbReN

Film thickness (nm)	8	10
ρ (μΩ·cm)	248	220
RRR	0.92	0.94
*D* (× 10^−4^ m^2^/s)	0.49	0.48
*T*_c_ (K)	5.31	4.61
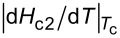 (T/K)	2.23	2.27
α = 3/(2*k*_F_ 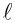 )	0.51	0.51
μ_0_*H*_p_(0) (T)	9.78	8.48
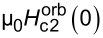 (T)	8.09	7.17
μ_0_*H*_c2_(0) (T)	8.12	6.38

**Figure 2:**
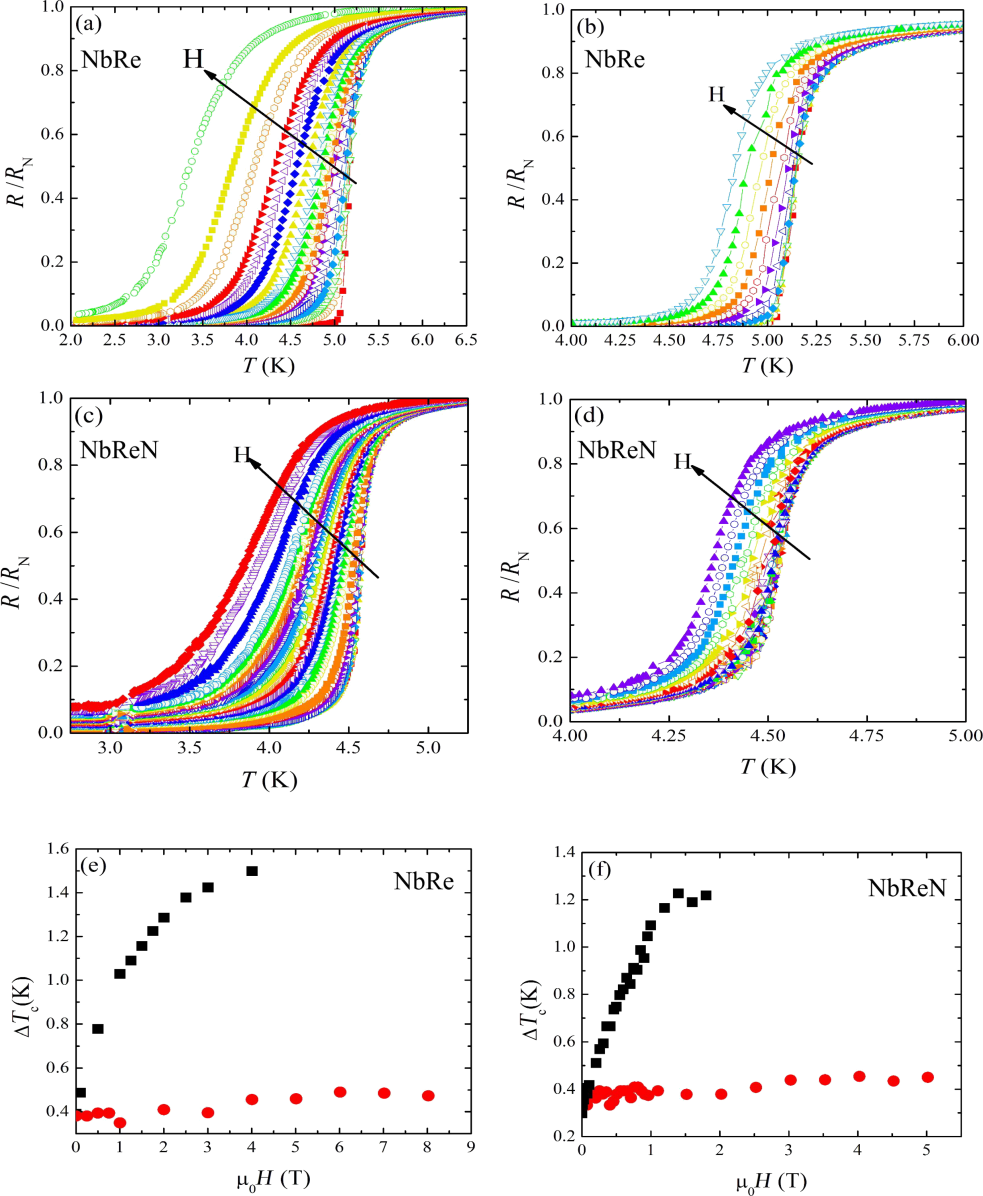
(a) Temperature dependence of the resistance of the NbRe microstrip in various magnetic fields in the perpendicular geometry. The curves have been measured for increasing magnetic fields (as indicated by the arrow) from μ_0_*H* = 0.003 to 4 T. (b) The same as panel (a) but in the parallel geometry. The field increases from μ_0_*H* = 0.003 to 8 T. (c) Temperature dependence of the resistance of the NbReN microstrip in various magnetic fields in the perpendicular geometry. The curves have been measured for increasing magnetic fields from μ_0_*H* = 0.001 to 3 T. (d) The same as panel (c) but in the parallel geometry. The field increases from μ_0_*H* = 0.007 to 5 T. (e, f) Δ*T*_c_ field dependence of (e) the NbRe and (f) the NbReN microstrip for the perpendicular (black squares) and parallel (red circles) geometries.

In Figure 2, the resistive curves of NbRe are shown for various values of *H* with the microstrip placed perpendicularly (Figure 2a) or parallely (Figure 2b) to the external field. The same quantities measured for NbReN are shown in Figure 2c and Figure 2d. In Figure 2e and Figure 2f, the field dependence of the width of the resistive transitions, Δ*T*_c_, is reported for NbRe and NbReN, respectively. We define 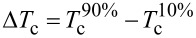, where 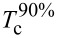
(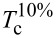) is the critical temperature obtained with the 90% (10%) *R*_N_ criterion. As it can be seen, when the samples are placed perpendicularly to the field the curves significantly broaden at high fields due to the entering of the vortices. However, the value of Δ*T*_c_ is similar for both materials. In contrast, in a parallel field, Δ*T*_c_ is almost constant in both cases.

**Figure 3:**
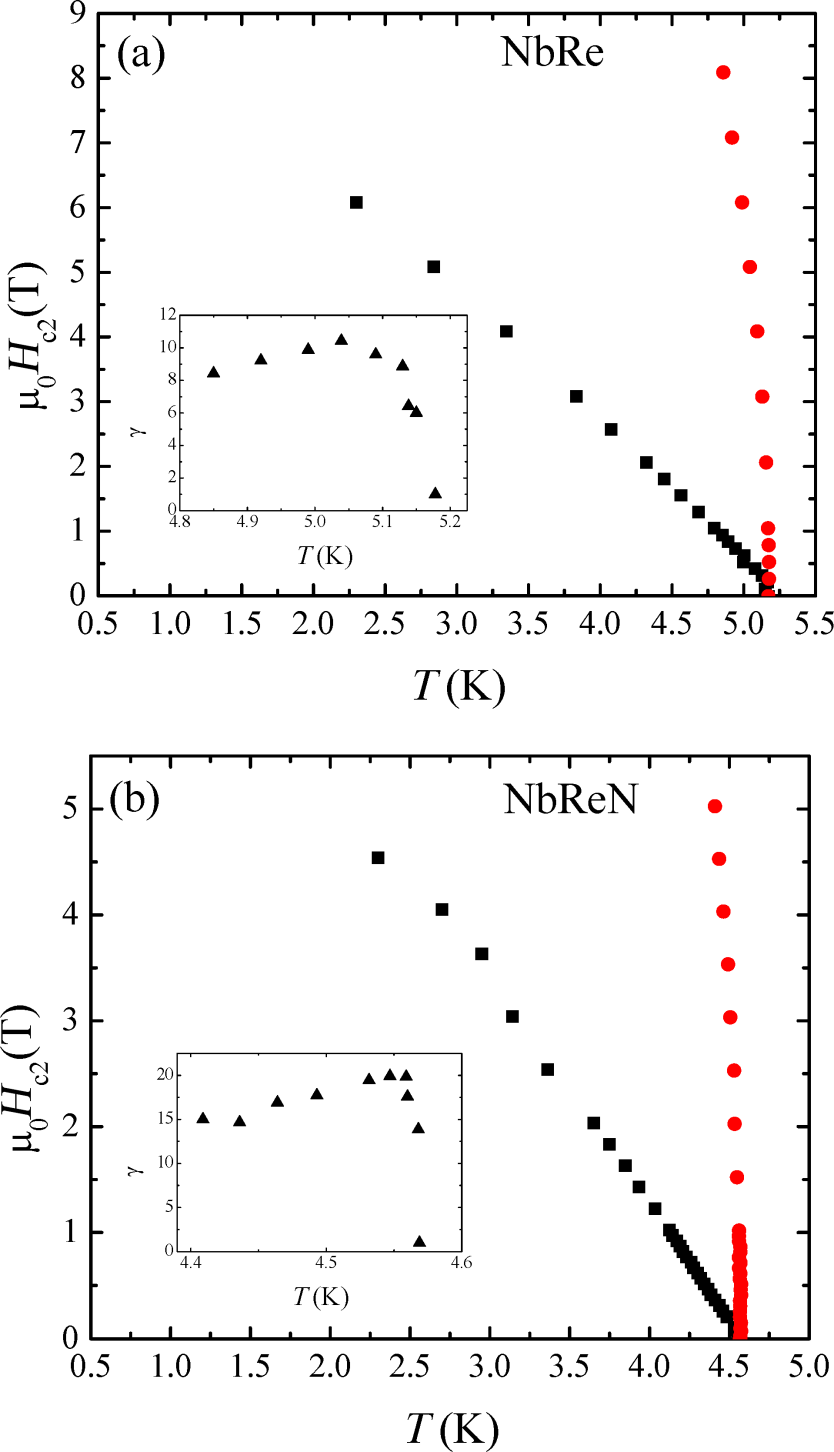
(a, b) *H*–*T* phase diagram of (a) the NbRe and (b) the NbReN microstrip. Black squares and red circles indicate the temperature dependence of *H*_c2⟂_ and *H*_c2∥_, respectively. The insets show the behavior of the anisotropy coefficient γ as a function of the temperature.

The temperature dependence of *H*_c2_ for NbRe and NbReN is displayed, respectively, in Figure 3a and Figure 3b, where both *H*_c2⟂_(*T*) and *H*_c2∥_(*T*) are reported. In the insets of Figure 3a and Figure 3b, the temperature dependence of the anisotropy coefficient γ(*T*) = *H*_c2∥_(*T*)/*H*_c2⟂_(*T*) is given for NbRe and NbReN, respectively. For both the materials, γ shows a nonmonotonic behavior with a fast increase followed by a smooth decrease when the temperature is lowered. However, for NbReN, γ is larger by a factor of almost two than for NbRe. We will comment on this point later on.

In the dirty limit and assuming that spin–orbit scattering is negligible with respect to spin-independent scattering, the temperature dependence of *H*_c2_ is given by the implicit equation [19]


**Figure 4:**
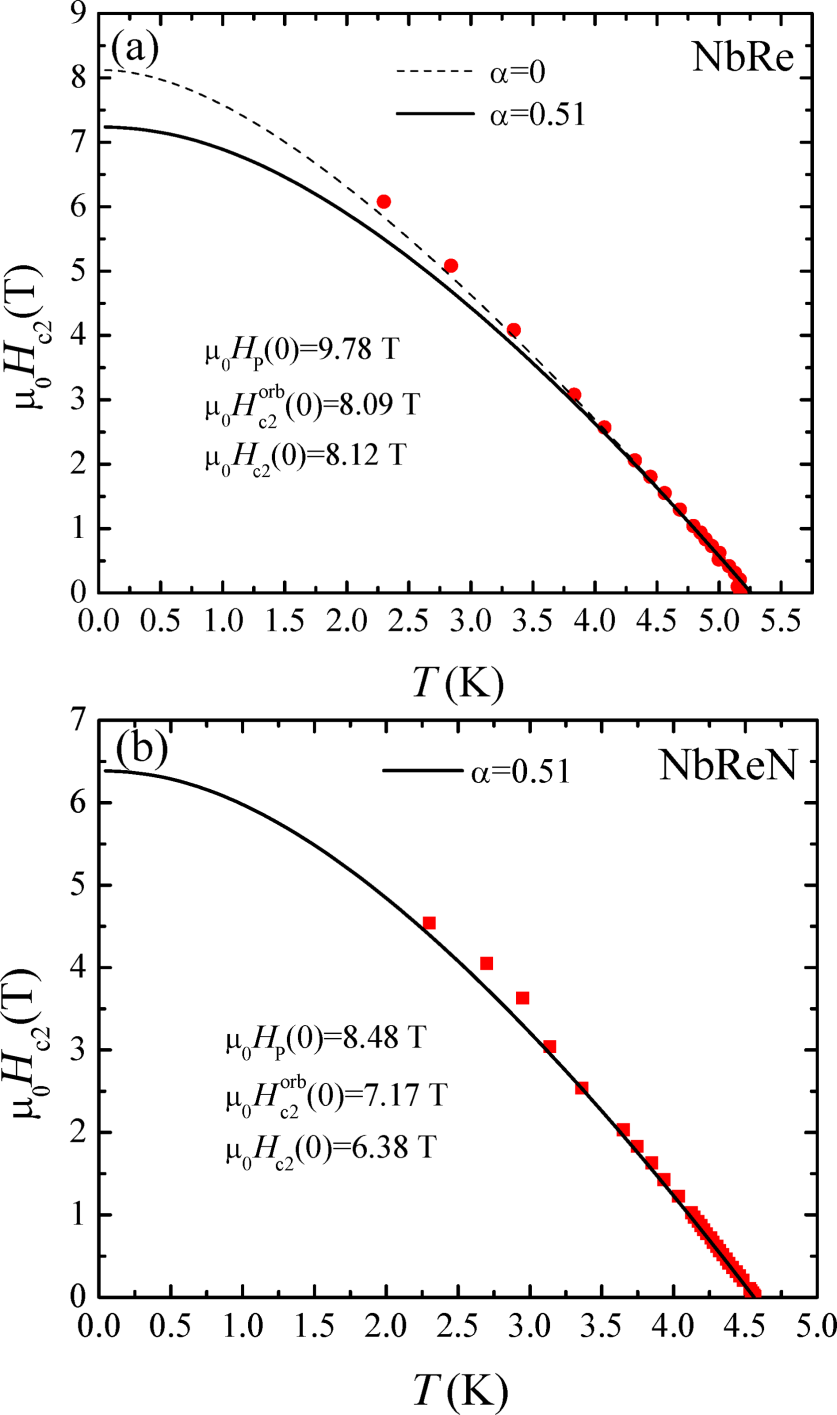
(a, b) Temperature dependence of the perpendicular upper critical field of (a) the NbRe and (b) the NbReN microstrip. The lines represent the WHH calculations. Details of the procedure are given in the text.

[1]

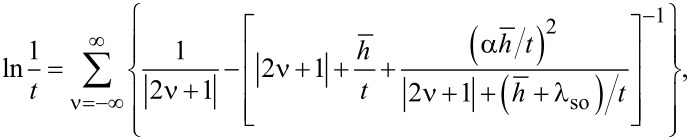



where *t* = *T*/*T*_c_, 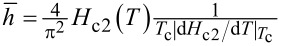. λ_so_ = ℏ/(3π*k*_B_*T*_c_τ_so_) with τ_so_ being the mean free time for spin–orbit scattering. We have used Equation 1 with λ_so_ = 0 to describe the experimental data for both materials using the measured values of *T*_c_ and the slope of *H*_c2⟂_ close to *T*_c_, which can be accurately determined via the many transition curves measured at very low fields. Moreover, the values of α for both materials have been obtained from the normal-state properties. Therefore, the WHH curves obtained from Equation 1 do not contain any fitting parameter [19,40]. All these quantities together with other superconducting and normal-state properties of the two materials are summarized in Table 1. In Figure 4 the perpendicular *H*–*T* phase diagram is reported for NbRe (Figure 4a) and NbReN (Figure 4b) microstrips together with the prediction given by the WHH theory. As far as NbRe is concerned, the experimental data are not described by the WHH theory considering α = 0.51, the value obtained from the normal-state properties (see solid line in Figure 4a). In fact, *k*_F_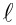 = 3*Dm*/ℏ [47], where *D* = 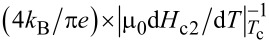 [48] is the quasiparticle diffusion coefficient and *m* is the mass of the electron. From the value of *D* reported in Table 1, we get *k*_F_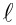 ≈ 1.3 and then α = 0.51. In contrast, data are very well reproduced by the WHH theory with α = 0, even though the points at the lowest temperature lay above the curve (see dashed line in Figure 4a). The value of the zero-temperature critical magnetic field [*H*_c2⟂_(0) = 8.12 T] is below *H*_p_(0) = 9.78 T, and since α = 0, the orbital limiting to *H*_c2⟂_ is the only contribution that should be considered. Our result, which is in line with other studies on *H*_c2⟂_(*T*) made on non-centrosymmetric materials in bulk forms [37,39,41], may suggest the presence of a triplet component of the order parameter. This result was even more evident in the case of polycrystalline NbRe samples, for which the experimental points in the *H*–*T* phase diagram stand well above the WHH line with α = 0 [14]. This result, interpreted in [14] as an indication of unconventional superconducting pairing, may be weakened in our case due to the poorer crystal quality of our disordered films. In the case of NbReN, the data are well described by the WHH theory with α = 0.51, as shown by the solid line in Figure 4b. In this case, the critical field is paramagnetically limited with the Pauli contribution that lowers the value of the critical field with respect to the pure orbital-limited case (α = 0). This suppression of the perpendicular critical field in conjunction with the steepest behavior of *H*_c2∥_ in the studied temperature range could also be the cause of the larger value of γ measured on the NbReN microstrip. Again, we ascribe this effect to the crystallographic properties of the films. Indeed, it is reasonable to suppose that the presence of the N atoms in the atomic cell may break the non-centrosymmetricity of the system, thus depressing the spin-triplet component of the order parameter. For this reason, *H*_c2⟂_ becomes paramagnetically limited [24,35]. In order to confirm these results, we are currently working on different experiments that may give more direct access to the order parameter in these systems. Regarding the aforementioned purpose, while in the case of NbRe it is now even more evident that films with larger crystallites are mandatory [7–8,12], detailed analyses of the NbReN crystal structure are still lacking and need to be performed.

## Conclusion

We have studied the *H*–*T* phase diagram for NbRe and NbReN microstrips. Despite the fact that the two materials show very similar morphological, normal-state, and superconducting properties, different results are obtained for the *H*_c2⟂_(*T*) behavior. In particular, while for NbRe the critical magnetic field is related to the orbital contribution, the Pauli limitation plays a relevant role in the case of NbReN. We correlate this result to the different microscopic properties of the two materials.
